# Factors Associated With Workplace and Interpersonal Trust in the Supervisory System of a Community Health Worker Programme in a Rural South African District

**DOI:** 10.34172/ijhpm.2021.03

**Published:** 2021-01-24

**Authors:** Tumelo Assegaai, Helen Schneider

**Affiliations:** ^1^School of Public Health, University of the Western Cape, Cape Town, South Africa.; ^2^South African Medical Research Council Health Services to Systems Unit, University of the Western Cape, Cape Town, South Africa.

**Keywords:** Workplace Trust, Interpersonal Trust, Community Health Workers, WBOT, Support, Supervision

## Abstract

**Background:** Key to effective supportive supervision, and ultimately performance of community health workers (CHWs), is the nature of relationships in the formal health system at the coal face of programmes. The central character and defining feature of effective relationships, in turn, is the ability to engender trust. This study describes factors associated with workplace and interpersonal trust, the relationship between the two sets of trust factors and how this shaped perceived performance of CHWs in ward-based outreach teams (WBOTs) in a rural South African district.

**Methods:** In the context of a wider study of supportive supervision of CHWs, factors recognised to be associated with trust in the literature were studied qualitatively in Ngaka Modiri Molema district, North West Province. Focus group discussions (FGDs) and individual interviews were conducted by the first author with CHWs (23), team leaders (12), facility managers (10) and middle managers (5). Interviews were recorded, translated and transcribed. Perceptions of trust factors associated with workplace and interpersonal trust were analysed thematically.

**Results:** The interviews revealed a climate of considerable workplace mistrust due to the perceived abandonment of the WBOTs programme by managers at all levels, and this affected support and supervision of WBOTs. However, there was a degree of variability and discretion in expressions of interpersonal trust at the coal face, leading to different perceptions of the competence and functionality of the WBOTs. Mistrust in the workplace and poor interpersonal relationships translated into low confidence in the ability of CHWs, which in turn compromised the performance of these teams.

**Conclusion:** The study contributes empirical evidence on how workplace trust factors impact on interpersonal trust factors and the possible implications of both sets of trust factors on perceived performance of CHWs. Wider trust in the health system have a significant bearing on interpersonal trust between CHWs and other players in the primary healthcare (PHC) system.

## Background

**Key Messages**

** Implications for policy makers**

- Supervision systems for community health worker (CHW) programmes should be judged by their ability to promote trust relationships between CHWs and other players.
- Workplace and interpersonal trust result from action at multiple levels of the health system.
- Manager support and resourcing of a programme are key factors in generating workplace trust, and impact on interpersonal trust relationships between CHWs and other players in the primary healthcare (PHC) system.
- Factors of trust and mistrust shape the perceived performance, confidence and motivation of CHWs.
- Supervision systems and trust relationships are key to strengthening and sustaining CHW programmes at scale.

**Implications for the public**

Community health workers (CHWs) act as a bridge between communities they serve and primary healthcare (PHC). In the South African setting, they typically provide health education, screening and follow-up support for health conditions such as tuberculosis and antenatal care. They are non-professional health workers with limited training and thus require effective support and supervision from the health system. Supportive supervision and performance of CHWs are linked to trust relationships with the health system and communities. This study seeks to supplement knowledge on ways to improve trust in the workplace and among front-line workers in order to strengthen the CHW programmes so that they can make a real impact on communities they serve.

Community health worker (CHW) programmes require effective support and supervision systems.^1,2^ Supervision of CHWs impacts on the performance of programmes as well as the ability of community-based services to coordinate with other players in the primary healthcare (PHC) system.^3,4^ Key to supportive supervision, and ultimately performance of CHWs, is the nature of relationships with both the formal health system and communities at the coal face of programmes.^2,5^ Health systems, more generally, can be viewed as fundamentally social systems of relationships which in part determine the performance of these health systems.^5-7^ The central character and defining feature of effective relationships, in turn, is the ability to engender trust.^6,8-11^ As pointed out: “health systems comprise a complex web of relationships whose overall functioning and performance is influenced by the institutions, particularly trust, that govern human behaviour.”^6^

Trust has been defined as “the optimistic acceptance of a vulnerable situation in which the trustor believes the trustee will care for the trustor’s interest.”^12^ Trust is relational and intangible and the basis of mutual dependability, confidence and management of risk in an organisation.^6,8^ In research on trust in healthcare settings, workplace trust generally refers to trust in the ‘system’ as well as interactions among health workers within the formal health system, while interpersonal trust tends to look at the interactions between health workers and users.^13-15^ Health workers who experience increased workplace trust have increased organisational commitment, healthy interactions and are more motivated to improve their performance and likely to be retained.^8,16,17^ Factors associated with trust in organisations are generally thought to include organisational and co-worker support, communication, respectful interactions, fairness, and competence.^7,8,10,13,15,17-19^ There has been limited consideration in the literature of the role of interpersonal trust *among* health workers in the health system nor of how workplace trust factors influence interpersonal trust factors in relationships at the coal face of the system.

In order to perform and deliver quality services, CHWs must be trusted and have trust in others.^7,14^ As intermediaries between communities and the health system, they are required to manage relationships in both directions.^11,19^ Navigating these relationships competently, with the limited training CHWs typically receive, requires effective systems of support and supervision.^7,18^ These systems constitute a range of direct and indirect relationships that ultimately impact on whether CHWs trust and are trusted. Yet, lack of trust in relationships between CHWs and health workers is frequently described, affecting the ability of CHWs to engage with communities.^7,11,18-21^ CHW programmes thus need to consider their social contexts and the mechanisms whereby trusting relationships could be ‘triggered’ to increase ‘social value’ and through this, performance.^7,9^

 The current CHW programme in South Africa was formalised in 2011 as part of a broader initiative to revitalise PHC. The programme is made up of ‘ward-based outreach teams’ (WBOTs) with 6 to 10 CHWs, led by a professional nurse called a ‘team leader.’ CHWs in WBOTs receive formal basic training and receive a monthly stipend. Team leaders are professional nurses mostly delegated from PHC facilities to supervise the team, and they report to the PHC facility manager. Each team is attached to a facility, operates within a municipality ward, and provides promotive and preventive services to individuals at household level. PHC facility managers have responsibility for oversight and support of teams.

Formal support from and integration into the local PHC system is a critical challenge facing national CHW programmes across the globe.^2,3,22-25^ In the South African context, the evidence shows that WBOTs are often placed at the bottom of a hierarchy where relationships with other PHC health workers are largely strained.^18,23,24,26,27^ In addition, findings from previous studies in the district found that PHC workers generally had a low degree of involvement in the WBOT supervisory and support system.^22,23^

 This study forms part of doctoral research assessing the supervision system of WBOTs conducted between 2017 and 2019 in Ngaka Modiri district, North West Province in South Africa. The study describes supervisory relationships from the perspective of the factors associated with trust and the implications of these trust factors for perceived performance.

## Methods

###  Design

A qualitative study of trust factors in relationships in the WBOTs programme was conducted as part of a 4-year engagement in the district by the first author as a doctoral candidate. This research has involved several phases of research informing this phase, including a prior quantitative social network analysis of relationships in the supervisory system.^22,23^

###  Conceptual Framework

**Figure 1:**
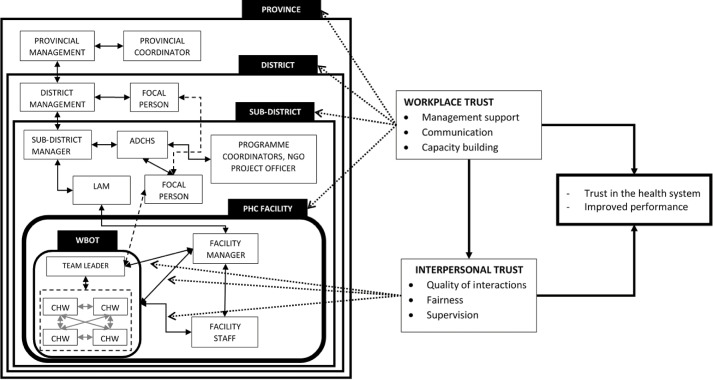
Conceptual Framework. Abbreviations: CHW, Community health worker; LAM, local area manager; WBOT, ward-based outreach team; ADCHS, assistant director - community health services; PHC, primary healthcare.

Figure 1 outlines the study conceptual framework, drawing on the workplace and interpersonal trust factors outlined by Gilson et al.^15^ We conceptualised workplace trust factors as referring to factors associated with health worker trust in the wider health system, and interpersonal trust factors as those associated with interactions amongst health workers. Factors influencing workplace trust identified from the literature included organisational support, communication and capacity building, while domains for interpersonal trust included communication, fairness and honesty.^7,8,10,13,17-19^ These relationships are embedded within political and social contexts, that shape workplace factors of trust, in turn influencing health worker morale, responsiveness and performance.

###  Study Setting

**Figure 2:**
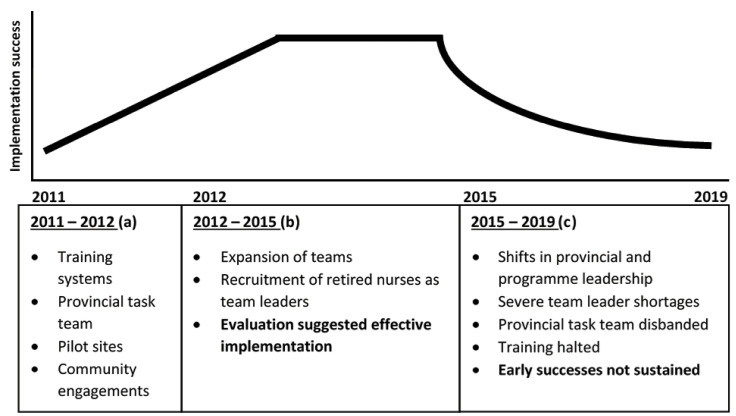
Functioning and Status of the Ward-Based Outreach Teams.

The study was conducted in 3 of 5 sub-districts of the Ngaka Modiri Molema district, one of 4 districts in the North West province. The district has one of the highest WBOTs coverage with 129 teams and has been considered a good performer in this regard, reflecting the rapid and effective early adoption of the WBOT programme in the Province as a whole. Initial stages of implementation of the WBOTs in the North West province included the setting up of a provincial Task Team; training of CHWs; establishment of pilot sites in all sub-districts; involvement of development partners to support implementation; extensive community engagement; the alignment with the district health information system; and an mHealth pilot (Figure 2 (a)).^28,29^ Around 2012/2013 (Figure 2 (b)), as more WBOTs were established, retired nurses were contracted, and nurses delegated from facilities to work as team leaders. An evaluation on the programme at the time described implementation as effective.^30^

However, these early successes were not sustained (Figure 2 (c)). From 2014/2015 onwards there were both changes in provincial and programme leadership and a growing political and fiscal crisis in the province. Contracts of retired nurses were not renewed, and facilities stopped delegating staff as team leaders, leading to a gross shortage of this immediate supervisory layer in established WBOTs. By 2019, 21 team leaders were serving the 129 WBOTs (personal communication district focal point). The provincial Task Team was disbanded, and training of team leaders and other relevant players linked to the WBOTs was halted. WBOT district level forums and meetings were absorbed into routine management processes.

 At the time of the study the provincial health department was ‘under administration’ of the national government stemming from a period of political instability and allegations of maladministration in the province.

 These contextual factors formed an important backdrop to the investigation of trust relationships.

As illustrated in Figure 1, a group of CHWs (usually 6) report to one team leader, although, as indicated, a dearth of professional nurses has meant that current team leaders supervise multiple WBOTs across different facilities. Team leaders are supervised by PHC facility managers where WBOTs are attached, supported by sub-district focal persons. Focal persons are professional nurses delegated to coordinate the WBOT programme at sub-district level, most of whom double up as team leaders to multiple WBOTs and report to the assistant director - community health services (ADCHS). Focal persons give team leaders and WBOTs feedback and updates and are supervised by the sub-district manager. PHC facility managers are supervised by local area managers (LAMs), responsible for a cluster of facilities. LAMs are supervised by a sub-district manager who reports to the district manager. Districts also have focal persons, and they report to the district manager for administrative matters and provincial specialists for clinical matters.^31^ A Healthcare Service Development Unit at provincial level, which reports to the provincial Deputy Director General provides support to WBOTs programme. Among other delegations, their role includes coordinating training for WBOT members with the districts and training centres, disseminating relevant information and updates to the district managers, and identifying and escalating district challenges to a WBOTs Provincial Steering Committee.

###  Participants and Sampling 

The main actors in the WBOT supervisory system, outlined above, formed the study population to describe trust factors, and included provincial managers, district managers, sub-district middle managers, focal persons, PHC facility managers, team leaders and CHWs. Participants were purposefully sampled from 3 (of 5) sub-districts where there was more than one team leader in place at the time of the study. In one of the sub-districts where an earlier study of social networks was conducted,^23^ participants were brought into the next phase. Participants sampled (50) for the study are as outlined in Table.

**Table:** Study Participants

**Level**	**Number of People**
LAM	3
PHC facility manager	10
Team leaders	12
CHWs	23
District middle manager	1
Provincial middle manager	1

Abbreviations: CHW, Community health worker; PHC, primary healthcare, LAM, local area manager.

###  Data Collection and Analysis

A semi-structured interview guide with open-ended questions was used for in-depth interviews with selected key informants through either focus group discussions (FGDs) or one-on-one interviews. The guide was piloted tested with one FGD interview, where it was found to be appropriate and the results included in the analysis. There were 7 FGDs (2 CHWs, 2 team leaders, 2 facility managers, 1 mixed group) and 3 individual interviews. The guide had 4 parts as prompts for the interviews. Participants were firstly given a diagramme outlining the web of actors and interactions involved in the functioning of WBOTs in the health system, and asked to give their views on the diagramme, including its accuracy (Supplementary file 1). The second part of the guide summarised results of the prior studies conducted by the researcher on the policy and practices of supervision and the social network analysis of supervisory relationships, respectively.^22,23^ This part of the guide was used to reflect on the nature of relationships in the supervision system. The third part of the guide asked respondents to reflect on the factors influencing the current supervision system of WBOTs, followed by specific probes for factors of trust.

To avoid the risk of social desirability bias, the guide did not directly ask about ‘trust’ but rather explored the factors of trust.^14,32^ Questions on workplace trust factors included how participants perceived the role of support and commitment of management towards WBOTs, processes of capacity building of CHWs and the nature of communication from higher levels of the system. For interpersonal trust, the factors explored were expressed confidence in the capacity of CHWs on the part of other actors, and the reported interactions among and between WBOTs members and PHC staff.

 The interviews were conducted between January and August 2019, in private and by the first author (TA) at participants’ place of work. All FGDs and 2 of the individual interviews were conducted in Setswana (local vernacular), while the rest were in English. The interviews were audio recorded, translated into English and transcribed. All the data was entered and coded through the ATLAS.ti 8 (ATLAS.ti Scientific Software Development GmbH, Berlin).

The analysis of the data was done deductively, based on the conceptual framework, using thematic analysis.^33^ The first author read through all the data transcripts to familiarise herself with the text, then identified codes, categorised these codes and developed themes that emerged from the text. The responses from the different sections of the guide were collated to provide a holistic picture of factors associated with trust and possible implications for the performance of WBOTs.

## Results

 Stemming from the growing management failures in the province, interviewee narratives expressed considerable lack of organisational trust and very variable relationships of trust and mistrust in interpersonal interactions at the frontline. We unpack each of these dimensions and the consequences for overall expressions of trust and confidence in the programme.

###  Factors Influencing Workplace Trust 

 Workplace trust factors explored were management support, capacity building and communication. With respect to management support, respondents described an environment of minimal support and apparent wholesale disengagement on issues relating to WBOTs from all layers of management in the health system.

 In the first instance, this was manifest in the absence of guidelines providing role clarification and expectations of various PHC players, with unclear lines of responsibility and a disconnect between players within sub-districts:

*“We have guidelines for each programme, and we are following those guidelines so that we can see if we are achieving targets or not. But with them [WBOTs] we don’t know if we are achieving or not” *(PHC facility manager).

*“There is confusion over roles. The PHC facility manager is given a role to manage the team leader, while there is also a focal person. There is also NGO [non-governmental organisation] project officer. […] in this situation supervision is weakened” *(PHC facility manager).

*“There is no communication between the LAM and the ADCHS [with respect to the WBOTs]. When you report challenges to the LAM it is as if it is not their baby”* (PHC facility manager).

*“There is no collaboration between the different programmes especially when they do outreach services. Facilities won’t even know there is outreach service in their back yards except when they [the programmes] need the CHWs, or equipment” *(LAM).

 Though the provincial structures continued to meet, there were no dedicated processes at district and sub-districts levels focusing on the WBOT programme. Communication within and across levels was poor, with relevant decisions taken at the provincial level not communicated to the sub-districts.

*“The district doesn’t take responsibility in making sure things like [feedback on meetings] reach the sub-districts”* (Manager).

 Sub-district line and programme managers were described as ‘uninterested’, as failing to take the programme seriously, and in some instances as actively hostile:

*“They do not take the programme seriously. They do not want to know what is happening. There was one time when we were here to get uniforms and [sub-district manager] asked us who we were [...] They don’t have interest in the programme”* (CHW).

*“…you mean the sub-district manager? We only see her when there are complaints. We can go a whole year without seeing her” *(CHW).

*“I still maintain it is lack of interest… That trophy [awarded to the WBOTs] sits in her office, we only see it passing by her office. We were never called to have it presented to us and to appreciate us. She has never said thank you to us for good work but she took credit for good work when we won”* (CHW).

*“[The sub-district manager] is ignorant and doesn’t like the programme*” (Focal Person).

*“Basically, they only come when the district says something is wrong go and check” *(PHC facility manager).

 There was a general expression of frustration in the lack of responsiveness by management to act on challenges related to the WBOTs.

*“… you can’t address the problem, you can only go and write a report, and that is if the person will read it. So going around […] and asking why, why, why, they lose trust in you” *(Manager).

*“Even if I raise issues, [management] keeps quiet. So I feel the only thing that is there for me is just to advise”* (Manager).

*“The LAM just walks in and out to another area” *(CHW).

*“Before, the LAM would come to the facility. Call all of us and we discuss our challenges. Nowadays it is not really happening”* (PHC facility manager).

 With respect to trust factor of capacity building, as alluded to, CHWs in the WBOT programme receive formal basic training. Although team leaders and facility staff occasionally provided on the spot guidance and training, continuing, in-service education of CHWs, considered key to performance and quality, was ad hoc and not systematically planned for, nor prioritised.

*“Those trainings do not exist at all”* (CHW).

###  Factors That Influence Interpersonal Trust 

 Factors of interpersonal trust examined were interactions of WBOTs with staff in PHC facilities, and experiences of fairness and support. While there was general consensus on (the problematic) factors of workplace trust, experiences of interpersonal trust factors at the coal face were more varied, leading to different perceptions of the competence and functionality of the WBOTs.

 Some facilities which had a better understanding of the WBOTs’ purpose and value, invested in the CHWs by developing their skills and inviting them to participate in facility activities. In these spaces, the WBOTs were considered to be effective and valued members of the PHC team.

*“My experience in general, they are like nursing assistants, they know their work*” (Team leader).

*“The facility manager can’t check each and everything like the baby books, so now we have a lot of hands to do that for you. The team I have is very dedicated, they will come and say here is a gap. Not reporting the person but thinking about the client and wanting to do what is right”* (PHC facility manager).

*“When you have problems in the households, and the team leader is absent, you can tell the facility to help you solve them” *(CHW).

 More often than not, however, facility workers did not understand the purpose and role of CHWs, and did not recognise them as part of the PHC system. In some facilities, CHWs were not well received, and facility workers described as harsh and dismissive. CHWs were actively excluded from facility processes, seen as pretending to be nurses and not allowed to use facility resources.

*“Some of the facility personnel feel the CHWs are not part of them. Because even with meetings, whether the CHWs attend or not the facility personnel do not care. Even if CHWs are absent, when the meeting starts, they call people individually to attend but not the CHWs”* (Team Leader).

*“Sometimes the facility manager asks you to assist in the facility […], you walk in innocently. The person will just say, these ones think they are better, they are taking our duties, they think they are nurses”* (CHW).

*“Even if they ask a clerk to make a copy for them, they shout at them “these things are not yours.” […] When I am in the facility it is better but when I am not there, they are ill-treated”* (PHC facility manager).

With respect to supervision, team leaders, where they were available, provided the main support and supervision to CHWs. As noted by one CHW, “*she assists us, we sit with her every month and we discuss our challenges*.”

With limited support and guidance from higher levels, facility staff were both unclear on roles and unable to adequately to support WBOTs. As one facility manager put it “*if you are not supported then your supervision becomes poor*.” Despite confusion on their roles, certain facilities were able to support CHWs in instances where no team leader was delegated, or if the team leader supervised multiple teams.

*“If they have problems, they are able to consult anyone in the facility. They ask, am I correct to do so and so on. So they get clarity from the facility manager*”(Team Leader).

###  Implications for Performance of the WBOT Programme

 In the facilities where there was inter-personal trust between players, the performance and ability of WBOTs was viewed positively. For example, in one such facility, staff members mentioned the following:

*“Just to add, I remember we had a high rate of malnutrition in the facilities. I think almost every week we would refer plus-minus three. It’s almost gone, it’s been a long time” *(acting LAM).

*“The other thing, if they do well, or the team performs very well, the facility tends to shine also”* (PHC facility manager).

 However, where interactions between CHWs and facility workers were described as poor, expressions of mistrust in the competence and integrity of CHWs were more common:

*“Sometimes they request us to assist with patient files, when the files get mixed up or someone can’t find a file, they say we have messed them up”* (CHW).

*“One old lady takes chronic medication, […] she has 2 mental health patients. There is a child on ARV [antiretroviral treatment], but this household is not registered, and we have CHWs in the community. It’s the third year. They are not working”* (PHC facility manager).

*“… sometimes I can tell the report was thumb sucked, so they don’t take the work seriously”* (PHC facility manager).

*“We are always told we are not working”* (CHWs).

 CHWs felt unrecognised and undervalued and expressed little belief in the future of the programme:

*“The truth is that our morale is low and one of the reasons is that we feel like we are not recognized” *(CHW).

*“It makes them feel small and doubt themselves in their work” *(Team leader).

*“One thing that bothers me is that this programme, I don’t see where it is going. Ever since joining I don’t see any future”* (CHW).

 Some managers described a programme in decline:

*“Some of them are even leaving the programme” *(Manager).

*“I am sad that the programme is dying on our watch”* (Manager).

 CHWs and programme managers alike thus recognised that declining material and moral support for the WBOTs programme from the provincial level were at the heart of poor trust relationships at district and facility levels.

## Discussion

This study examined factors of trust and mistrust in a CHW programme at sub-national level. It supplements existing work on trust relationships of CHWs in health systems,^7,11,18,19^ by describing how factors associated with workplace trust impact on interpersonal trust factors and how these shape the perceived performance of WBOTs.

The findings complement existing evidence that support and supervision roles at multiple levels need to be addressed for sustained implementation of CHW programmes at scale.^23,34^ Although the North-West Province was an early adopter of the WBOTs, an unfavourable political and economic context in the subsequent years of implementation led to the loss of management commitment displayed at the onset across all levels.^28^ There was limited accountability and responsibility from senior management towards the programme, and therefore poor coordination of the programme in a manner that instilled confidence and trust in front-line workers and WBOT members. Participants overall did not believe the supervisors acted in their interest, and there was significant mistrust in management and among actors in the district.

The vulnerability of trust relationships in CHW programmes to wider system failings has been documented elsewhere. A study in Malawi found that limited management support and engagement resulted in low trust in CHWs in rural areas,^19^ echoed in other African countries^7^ and in Guatemala.^35^

In this study, general organisational mistrust set the conditions for interpersonal mistrust and perceptions of low competency and functionality of the programme. Despite this, some frontline players were able to swim against the tide, expressing their agency by building interpersonal trust and confidence in the WBOTs. This observation provides valuable lessons on how to nurture resilience in the health system through positive relationships, as opposed to a more common focus of health system strengthening on compliance with standards and targets.^7,13,36^ As found in other studies, relationships and trust are linked to performance and motivation of CHWs.^6,7,10,11,13,15^

In this study, experiences of interpersonal trust varied and so were perceptions about the CHWs. In facilities where it was thought the roles and functions of CHWs were not clear, PHC workers were perceived to have no confidence in the competencies of CHWs, treated CHWs unfairly and the quality of their interactions was poor. In facilities where CHWs roles and functions were understood and appreciated, CHWs had better interactions with health workers, they were capacitated, supported and supervised. Interpersonal trust relationships depend on how health workers perceive CHWs’ ability to render appropriate services,^18^ and affect interdisciplinary team work and collaboration.^10,11^

In order to build trust, it is also important to resource CHW programmes with sufficient funding, human resources and supplies.^37^ It is also necessary to strengthen programme governance systems and processes from province to the coal face of delivery.^38^ In doing so, there is the opportunity to learn from the people in the front line who managed to keep the CHW programme going, despite the existing challenges. Sharing such experiences through the system would complement top-down with bottom up processes of learning on enablers of trusting relationships.

Although context and issue specific, the study contributes insights into health systems supervisory relationships and their implications for performance.^6,7^ The relational lens of trust provides a useful framing for looking at functionalities and dysfunctionalities of broader support systems, including supportive supervision, for front line workers.^6,18,19^ It speaks to the ‘people’ aspects of health systems, how their relationships are shaped, and how they experience the system, as opposed to objective criteria, like the ratio of supervisors to CHWs, the presence of a manual, or checklists of resources.^7,13^

 A limitation to the study is that the first author has a prolonged engagement in the study site, and this may have posed a potential bias in understanding and analysing findings. The involvement of the second author as an external player and critical mirror helped to minimise this. On the other hand, the author’s long association with the programme enabled her to contextualise findings in trends over time. Another limitation was that the study was confined to one district. The specific experiences of this district should not be read as representative of the whole province or country.

## Conclusion

 The study contributes to an important body of work by providing empirical evidence on how factors of workplace trust impact on those of interpersonal trust and possible implications of both forms of trust factors on perceived performance of CHWs. Wider trust/mistrust in the health system has a significant bearing on factors associated with interpersonal trust between CHWs and other players in the PHC system. Relationships of trust are a key outcome of effective supervision and performance in CHW programmes. It is important to design and facilitate supervision systems in ways that promote relationships and generate trust between CHW programmes and the health system to strengthen performance and sustain the programme at scale.

## Acknowledgements

 The work reported herein was made possible through funding by the South African Medical Research Council through its Division of Research Capacity Development under the Bongani Mayosi National Health Scholars Program from funding received from the South African National Treasury. The content hereof is the sole responsibility of the authors and do not necessarily represent the official views of the SAMRC or the funders.

## Ethical issues

 Ethical approval for the study was obtained from the University of the Western Cape Research Ethics Committee (registration number BM/17/3/3) and the North-West Provincial Department of Health Research Ethics Committee. All participants were provided with an information sheet and participants were given time to familiarise themselves with the topic and purpose of the study and then given time to ask questions. Participants provided signed written informed consent to be interviewed and audio recorded.

## Competing interests

 Authors declare that they have no competing interests.

## Authors’ contributions

 The study design was developed by TA and HS. TA collected the data and performed the analysis under the supervision of HS. TA drafted this article and both authors revised the manuscript.

## Disclaimer

 The views expressed in the submitted article are those of the authors and not their funders.

## Authors’ affiliations

^1^School of Public Health, University of the Western Cape, Cape Town, South Africa. ^2^South African Medical Research Council Health Services to Systems Unit, University of the Western Cape, Cape Town, South Africa.

## Supplementary files


Supplementary file 1 contains the role players and relationships in the supervision of WBOTs.
