# Supplementary figures and images for: Factors Associated With Workplace and Interpersonal Trust in the Supervisory System of a Community Health Worker Programme in a Rural South African District
Source: Int J Health Policy Manag. 2021 Jan 24;11(1):31–8. doi: 10.34172/ijhpm.2021.03 (PMC9278395; doi:10.34172/ijhpm.2021.03)

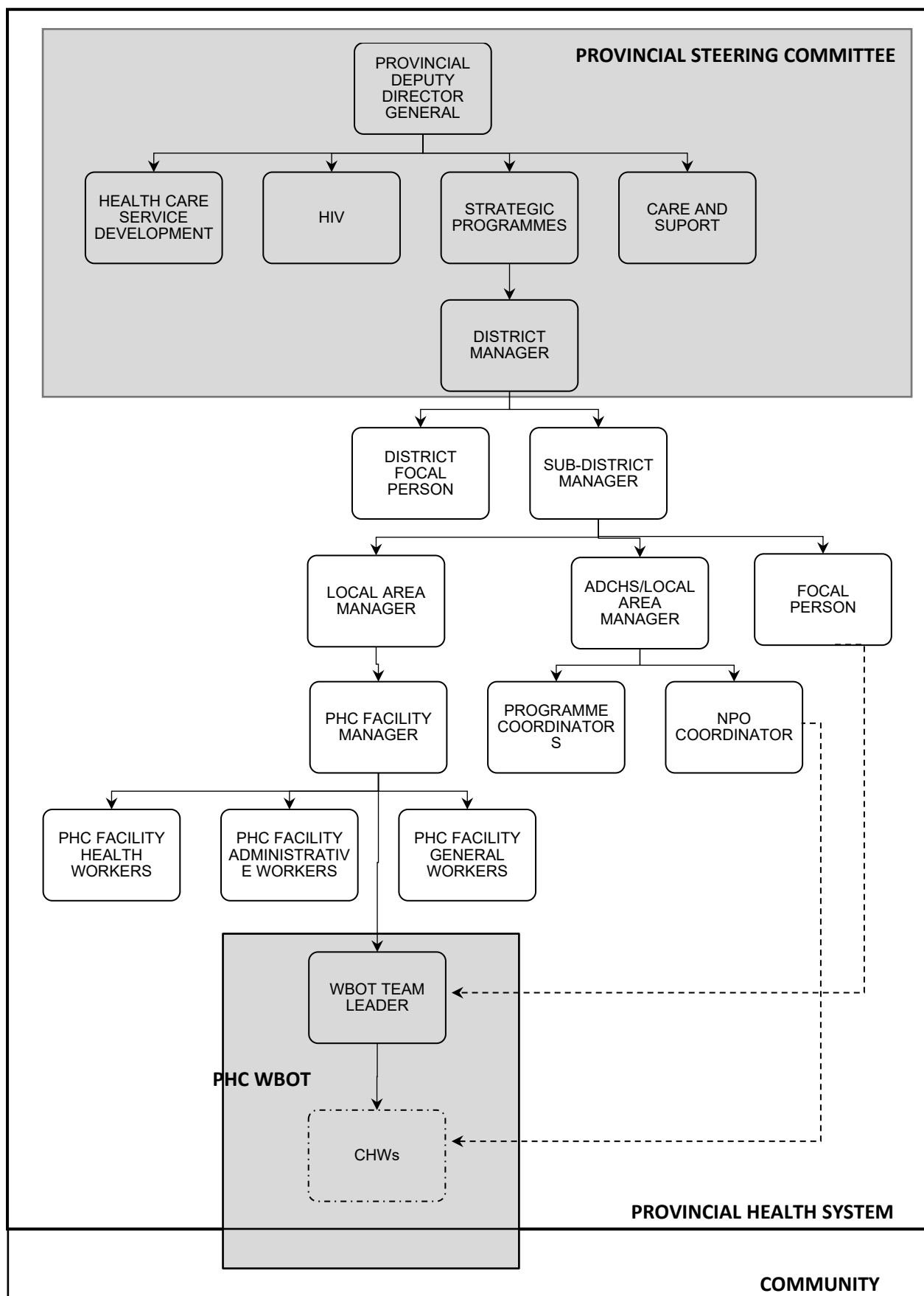

Supplement: Supplementary file 1 — contains the role players and relationships in the supervision of WBOTs. [file ijhpm-11-31-s001.pdf]
